# Genetic Variation in *OAS1* Is a Risk Factor for Initial Infection with West Nile Virus in Man

**DOI:** 10.1371/journal.ppat.1000321

**Published:** 2009-02-27

**Authors:** Jean K. Lim, Andrea Lisco, David H. McDermott, Linda Huynh, Jerrold M. Ward, Bernard Johnson, Hope Johnson, John Pape, Gregory A. Foster, David Krysztof, Dean Follmann, Susan L. Stramer, Leonid B. Margolis, Philip M. Murphy

**Affiliations:** 1 Molecular Signaling Section, Laboratory of Molecular Immunology, National Institute of Allergy and Infectious Diseases, National Institutes of Health, Bethesda, Maryland, United States of America; 2 Section on Intercellular Interactions, Laboratory of Cellular and Molecular Biology, National Institute of Child Health and Human Development, National Institutes of Health, Bethesda, Maryland, United States of America; 3 Comparative Medicine Branch, National Institute of Allergy and Infectious Diseases, National Institutes of Health, Rockville, Maryland, United States of America; 4 Illinois Department of Public Health, Division of Laboratories, Chicago, Illinois, United States of America; 5 Colorado Department of Public Health and Environment, Denver, Colorado, United States of America; 6 Biostatistics Research Branch, National Institute of Allergy and Infectious Diseases, National Institutes of Health, Bethesda, Maryland, United States of America; 7 American Red Cross, Gaithersburg, Maryland, United States of America; The Rockefeller University, United States of America

## Abstract

West Nile virus (WNV) is a re-emerging pathogen that can cause fatal encephalitis. In mice, susceptibility to WNV has been reported to result from a single point mutation in *oas1b*, which encodes 2′–5′ oligoadenylate synthetase 1b, a member of the type I interferon-regulated *OAS* gene family involved in viral RNA degradation. In man, the human ortholog of *oas1b* appears to be *OAS1*. The ‘A’ allele at SNP rs10774671 of *OAS1* has previously been shown to alter splicing of *OAS1* and to be associated with reduced OAS activity in PBMCs. Here we show that the frequency of this hypofunctional allele is increased in both symptomatic and asymptomatic WNV seroconverters (Caucasians from five US centers; total n = 501; OR = 1.6 [95% CI 1.2–2.0], *P* = 0.0002 in a recessive genetic model). We then directly tested the effect of this SNP on viral replication in a novel *ex vivo* model of WNV infection in primary human lymphoid tissue. Virus accumulation varied markedly among donors, and was highest for individuals homozygous for the ‘A’ allele (*P*<0.0001). Together, these data identify *OAS1* SNP rs10774671 as a host genetic risk factor for initial infection with WNV in humans.

## Author Summary

When humans are exposed to infectious agents, the outcome may vary: some remain uninfected, some who become infected remain asymptomatic, and symptomatic individuals may develop clinical manifestations that vary in number and severity. Both host and environmental factors are thought to influence outcome. Here we show that a specific human mutation that results in reduced function in a known antiviral gene named *OAS1* is associated with increased rate of infection with West Nile virus, a member of the flavivirus family. *OAS1* codes for a component of the type I interferon signaling system; its homologue in mice has been shown previously to be important for flavivirus susceptibility. Individuals carrying two copies of the hypofunctional variant of *OAS1* were found more frequently than expected among both asymptomatic and symptomatic WNV-positive individuals. To investigate further, we developed a novel model of WNV replication using human tonsil tissue in which high variability in replication was observed among donors. We found that the highest virus accumulation occurred in donors with two copies of the hypofunctional *OAS1* variant. Together these data suggest that OAS1 activity may influence the probability of initial infection, but not the severity or symptomatic quality of infection, after WNV exposure in man.

## Introduction

West Nile virus (WNV) is a re-emerging flavivirus transmitted by mosquitoes to several species of birds. Humans and several other mammalian species may be accidental dead-end hosts. First isolated in Uganda from a febrile woman in 1937 [1], WNV has caused sporadic outbreaks in the Middle East, Africa, Western Asia, Europe, and Australia. In the Western Hemisphere, it was first isolated from a patient during an outbreak of meningoencephalitis in New York in 1999 [2],[3],[4]. Since then it has rapidly spread across the United States into Canada and Central and South America, and has caused annual outbreaks of disease. Through November 18, 2008, there have been 28,906 US laboratory-confirmed symptomatic WNV-seropositive cases reported to the Centers for Disease Control and Prevention (CDC), with 1121 (3.9%) WNV-induced deaths (www.cdc.gov). In addition to human infections, the virus has caused significant morbidity and mortality in birds and horses.

In the US, seroprevalence is ∼3% in the general population and 20–30% of infected individuals have been estimated to become symptomatic [5]. Clinical manifestations include West Nile fever (WNF) and WNV-induced neuroinvasive disease (WNND), which manifests primarily as meningitis and encephalitis [6]. To date, no specific antiviral agents or vaccines have been approved by the FDA for human WNV infection, and treatment is supportive. Survivors of WNND may develop long-term neurologic sequelae.

Viral infections in man are typically controlled in part by early induction of type I interferon (IFN), which initiates a cascade of innate mechanisms that impair virtually all aspects of the viral life cycle. Genetic studies in mice have identified several type I IFN-regulated anti-viral effector pathways that control WNV replication, including the double-stranded RNA-dependent protein kinase (PKR) mechanism of protein synthesis inhibition, and the 2′–5′ oligoadenylate synthetase (OAS) pathway of RNaseL-mediated RNA degradation [7]. OAS enzymes catalyze the synthesis of 2′–5′-linked oligoadenylates (2–5A) from ATP, which can then bind and activate latent RNaseL resulting in the degradation of host and viral RNAs [8].

Targeted gene disruption in mice has revealed a critical role for the type I IFN Receptor, PKR and RNaseL for survival following WNV infection [7],[9]. A critical role for OAS emerged from genetic analysis of the mouse locus *flv*, which determines in an autosomal dominant manner susceptibility to WNV and other flaviviruses [10],[11]. *Flv* has recently been mapped by two groups working independently [12],[13] to a missense mutation in the 2′–5′ oligoadenylate synthetase 1b (*oas1b*) gene, which results in a truncated protein. How this mutation actually works has not yet been fully delineated. It clearly is not redundant with the multiple other *oas1* genes found in the mouse genome [14]. However, there is evidence that protection may not actually be mediated through RNase L [15], and it is not yet known whether the *oas1b* gene product is enzymatically active [16]. The genetic data suggesting a role for *oas1b* in protection against WNV in mice are supported by *in vitro* experiments showing that WNV replication in cells expressing wild-type *oas1b* is less efficient than in cells expressing the truncated form [17],[18]. Furthermore, genetic knock-in of the resistant *oas1b* allele into a susceptible mouse strain resulted in resistance to Yellow Fever Virus, a related flavivirus [19]. WNV susceptibility has not been reported in this knock-in mouse. Taken together, the combined *in vivo* and *in vitro* data suggest an important role of *oas1b* in innate resistance to flavivirus infection in the mouse.

Thus, we hypothesized that polymorphisms in the human ortholog of *oas1b*, *OAS1* [14], could influence outcome in humans exposed to WNV. Established risk factors for human WNV disease include age, immunosuppression, and genetic deficiency in the chemokine receptor/HIV-1 coreceptor *CCR5*, due to homozygous inheritance of the defective allele *CCR5Δ32* [20],[21]. Despite the strong epidemiologic association with *CCR5Δ32*, only ∼4% of Caucasian symptomatic WNV-infected individuals can be accounted for by CCR5 deficiency, indicating that other genetic risk factors for WNV disease may exist. A small case-control study (n = 27 cases) failed to find evidence of association of severe WNV disease with two *OAS1* SNPs [22]. Instead, the investigators found a strong association with a silent variant (rs3213545) in *OAS-Like* (*OASL*), a paralogue of *OAS1* located approximately 8 megabases away that has not been found to encode a protein with OAS activity [8]. In this report, we attempted to validate the *OASL* association with WNV, but could not. Instead, we found a consistent association of an *OAS1* SNP and infection with WNV from samples collected from five independent US centers, and with WNV replication in human lymphoid tissue *ex vivo*.

## Results

### Structure and function of *OAS1* SNP rs10774671

**Figure 1:**
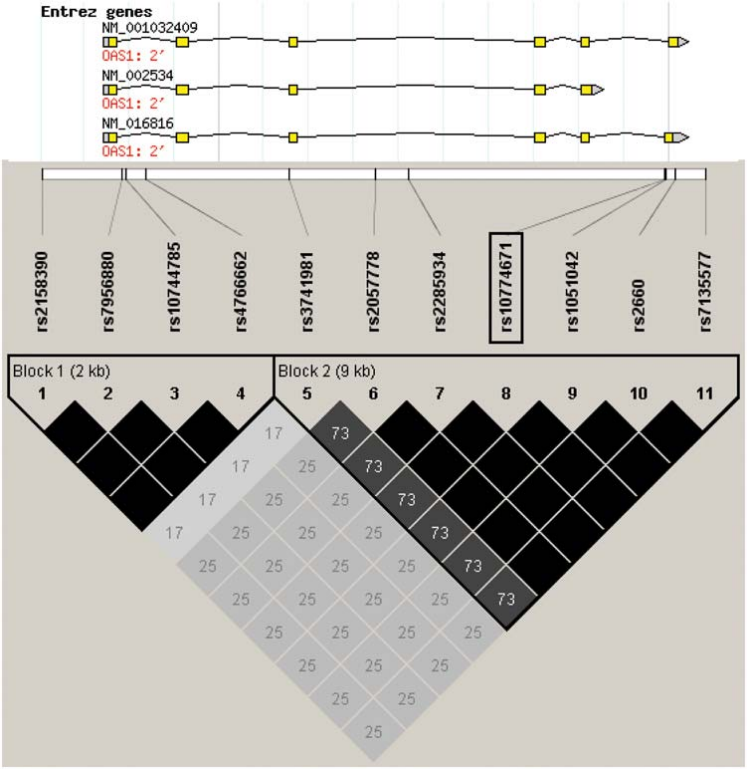
Haplotype block structure of the human *OAS1* gene in the CEPH cohort. The location of rs10774671 (boxed) and the surrounding SNPs in and around the *OAS1* gene are shown. LD block structure of the *OAS1* gene found in the CEPH cohort was analyzed using Haploview version 4.0. Pairwise r^2^ values are scaled in gray with higher r^2^ values shown in darker color; black indicates an r^2^ = 1.

To investigate the role of the OAS system in controlling outcome after WNV exposure in man, we scanned the *OAS1* gene for known polymorphisms that occur in this region. Unlike the mouse genome, where 8 copies of *oas1* have been identified (*oas1a-h*), there is only one copy of *OAS1* in the human genome, although alternative splicing gives rise to several isoforms [14]. All *OAS1* splice variants include exons 1–5, but vary with regard to downstream exons, and thus produce proteins of various sizes, including the p42, p44, p46, p48, and p52 forms [14],[23]. We identified common SNPs in the *OAS1* gene region in the CEPH cohort using data from the International HapMap Project (www.hapmap.org). Human *OAS1* is one of three genes in the *OAS* cluster, ordered *OAS1*, *OAS3* and *OAS2* on chromosome 12 [24]. As shown in Figure 1, at least eleven SNPs in the *OAS1* region have been found to cluster into two blocks of linkage disequilibrium (LD). The first block contains four SNPs, three of which are found within the first intron (rs7956880, rs10744785, and rs4766662) and one of which (rs2158390) is found in the 5′UTR. The second block contains seven SNPs, with rs3741981 in exon 3; rs2057778, rs2285934 and rs2285934 in intron 3; rs10774671 located at the last nucleotide in intron 5; rs1051042 and rs2660 in exon 7; and rs7135577 in the 3′UTR. The SNPs found in block 1 are not in coding regions. The six SNPs in block 2 excluding rs3741981 are in tight LD (Figure 1, the black region in block 2) and form two haplotypes that have been previously shown by Bonnevie-Nielsen and colleagues to be highly associated with the level of OAS enzymatic activity measured in peripheral blood mononuclear cells [23]. These investigators concluded that rs10774671 is most likely to be the functional SNP in this block because it sits at the last nucleotide of intron 5 in the *OAS1* gene and serves as a splice acceptor site for exon 7. The G allele is predicted to allow splicing to occur resulting in the production of a p46 form with high enzymatic OAS activity. The A allele is predicted to prevent splicing at this site; instead, splicing occurs further downstream, resulting in two other forms, designated p48 and p52 associated with lower OAS enzymatic activity. Heterozygotes were reported to have intermediate OAS activity [23]. Although other splice variants of this gene exist, including p42 and p44, the splice variants that arise from the use of exon 7 (p46, p48, and p52) are controlled by rs10774671 [23],[24]. Thus, this polymorphism is a biologically plausible genetic probe to investigate the role of OAS1 in human WNV disease.

### Study participants and genotype analysis of *OAS1* SNP rs10774671

To test whether this SNP is associated with WNV infection, we obtained serum or plasma from five independent US sources of Caucasian WNV+ individuals (n = 501). We collected symptomatic WNV-seropositive patient samples (n = 331) from the following state public health departments and outbreak years: Arizona (2004), California (2005), Colorado, (2003) and Illinois (2005–6) as detailed in Table 1. These samples were from individuals who sought medical attention and where WNV was considered in the differential diagnosis because of compatible signs and symptoms (fever, meningitis, encephalitis) and confirmed by serologic testing. We also obtained asymptomatic WNV-seropositive blood donor samples (n = 170) from the American Red Cross (ARC) national blood supply screening program, identified as WNV nucleic acid amplification test (NAT) reactive and confirmed by serologic testing. These individuals remained symptom-free, according to self-assessment in a follow-up questionnaire, for at least 2 weeks post-donation [25],[26].

**Table 1:** Characteristics and genotypes of self-reporting Caucasian study subjects.

	AZ WNV+	CO WNV+	CA WNV+	IL WNV+	ARC WNV+	Combined WNV+	ARC WNV−	RBD WNV−	Combined WNV−
Number	135	72	87	37	170	501	192	360	552
Mean age (y)	57±18	50±18	54±18	54±19	52±15	53±17	49±15	55±13	
% Male sex	52.6	43.1	51.7	62.2	72.9	58.7	56.3	58.3	56.8
% WNF^a^	36.3	22.2	62.1	–	0	36	–	–	–
% WNND^a^	60	77.8	31	–	0	49.5	–	–	–
% Unspecified^a^	3.7	0	6.9	100	0	14.5	–	–	–
% Mortality (n)	4.4 (6)	1.4 (1)	6.9 (6)	–	0	4.4 (13)^b^	–	–	–
% *OAS1* AA (n)	51.1 (69)	48.6 (35)	47.1 (41)	51.4 (19)	48.8 (83)	49.3 (247)	40.6 (78)	36.7 (132)	38.0 (210)
% *OAS1* AG (n)	38.5 (52)	44.4 (32)	35.7 (31)	48.6 (18)	38.2 (65)	39.5 (198)	46.2 (89)	46.4 (167)	46.4 (256)
% *OAS1* GG (n)	10.4 (14)	6.9 (5)	17.2 (15)	0.0 (0)	12.9 (22)	11.2 (56)	13.0 (25)	16.9 (61)	15.6 (86)
% *OAS-Like* TT (n)	6.7 (9)	15.3 (11)	6.9 (6)	10.8 (4)	10.6 (18)	9.6 (48)	12.0 (23)	8.9 (32)	10.0 (59)
% *OAS-Like* CT (n)	44.4 (60)	44.4 (32)	39.1 (34)	56.8 (21)	40.0 (68)	42.9 (215)	41.7 (80)	41.9 (151)	41.8 (245)
% *OAS-Like* CC (n)	48.9 (66)	40.3 (29)	54.0 (47)	32.4 (12)	49.4 (84)	47.5 (238)	46.4 (89)	49.2 (177)	48.2 (281)

a Cases from US states were classified into either West Nile fever (WNF), West Nile neuroinvasive disease (WNND), or unspecified disease categories by physician interview. All samples from ARC experienced no symptoms as assessed by questionnaire ≥2 weeks post-donation.

b Mortality data do not include Illinois for which clinical outcome was not provided. RBD, random blood donors; AZ, Arizona; CO, Colorado; CA, California; IL, Illinois; ARC, American Red Cross; WNV+, WNV-seropositive individuals; WNV−, WNV-seronegative controls. All groups were in Hardy-Weinberg equilibrium. Genotypic frequencies where the total does not equal 100.0% are due to rounding imprecision.

For comparison, we obtained two groups of US Caucasian control samples (n = 552). The first was comprised of healthy US Caucasian random blood donors (RBD) collected prior to the introduction of WNV into the US in 1999 (n = 360). The second consisted of healthy US blood donors collected by the ARC who were identified during routine blood screening as WNV false positives (initial reactivity by WNV NAT that could not be replicated and WNV-seronegative (n = 192)).

We limited the analysis to self-reporting Caucasian individuals because the allele frequency of the *OAS1* rs10774671 SNP varies according to race (www.ncbi.nlm.nih.gov/projects/SNP/). In both cases and controls, the overall genotyping success rate was >96.0%. 156 patient samples were re-tested using a second genotyping method with 100% concordance. Within each group (WNV+ and control), the *OAS1* genotypes were in Hardy-Weinberg equilibrium (HWE; *p* = 0.51 and *p* = 0.93, respectively). In our control samples (n = 552), the frequency of the *OAS1* rs10774671 AA genotype shown in Table 1 (38.0%) was consistent with the observed AA genotypic frequency in other published Caucasian cohorts representing over 5000 individuals predominantly from Europe (36.8–41.1%) [27],[28],[29] as well as the published report in the CEPH cohort in the HapMap Project (39%, n = 59; www.hapmap.org).

### *OAS1* rs10774671 AA genotype is elevated in WNV-infected individuals

**Figure 2:**
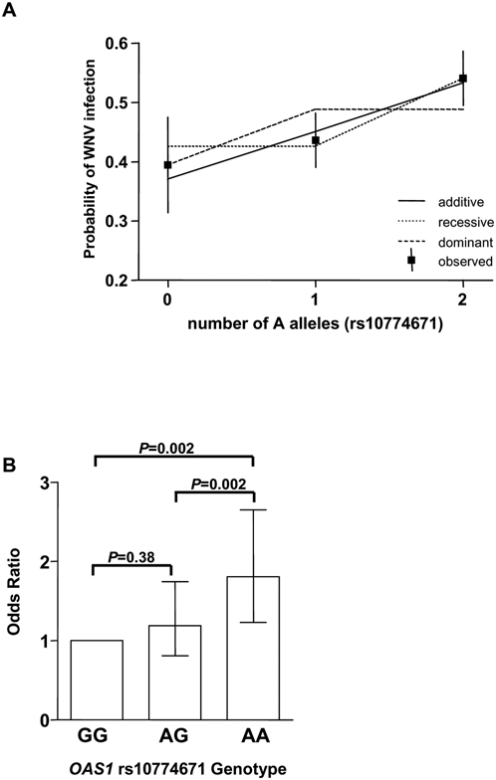
*OAS1* allele dosage effect on risk of WNV infection. (A) A plot of the estimated probabilities of being WNV-positive as a function of the number of copies of rs10774671 A allele under three different genetic models. The solid line is the additive model; the dotted line is the recessive model; the dashed line is the dominant model. Also graphed are the observed proportions of WNV cases (solid squares) along with 95% CI (solid vertical lines). (B) The allele dosage effect of the A allele for SNP rs10774671 was examined using GG homozygous individuals as a reference genotype. OR (bars) were calculated, and 95% CI shown (lines) were determined for all study subjects. Chi-square test was used to calculate *P* values (shown).

A genotypic contingency analysis (3×2) for *OAS1* rs10774671 revealed a statistically significant association when WNV-positive samples were compared to controls (*p* = 0.0008). In our analysis, we considered the dominant, recessive and additive genetic models (Table 2), and all were statistically significant (*p*<0.05). Using the recessive model when all cases were grouped together, the AA genotype was significantly greater in WNV-positive subjects than in controls (Odds Ratio (OR) = 1.6 [95% Confidence Interval (CI) 1.3–2.1, *p* = 0.0001; Table 2). A similar trend in OR was observed in each of the 5 centers when considered separately, ranging between 1.5 and 1.7. Analysis of the data using the additive model also showed a highly significant association (OR = 1.4 per allele [95% CI 1.2–1.7], *p* = 0.0003; Table 2) indicating that having more A alleles was significantly greater in WNV-positive individuals than in controls. The OR for each center when considered separately was less consistent in this model, spanning between 1.2 and 2.0. To determine which model fits the data best, we analyzed the predicted probability of WNV infection by copies of A allele under the three models (Figure 2A). Superimposed are the observed proportions of WNV-positive individuals by copies of A (square boxes) along with the 95% CI. By comparing the observed to predicted proportion of cases by number of alleles using chi-square goodness-of-fit tests, we determined that the recessive and additive models are approximately equally supported by the data, with the dominant model having a decidedly poorer fit. We also analyzed each genotype separately, using GG as the reference genotype, since the G allele is the ancestral allele (from published *Pan troglodytes* sequence data, www.ncbi.nlm.nih.gov). As shown in Figure 2B, the strongest association with WNV infection was observed when AA was compared to the GG genotype (OR = 1.8, 95% [CI] 1.2–2.7, *p* = 0.002). There was also a statistically significant increase between the AG and AA genotypes (OR = 1.5 95% [CI] 1.2–2.0, p = 0.002). The comparison between the GG and AG genotypes was not statistically significant (OR = 1.2, 95% [CI] 0.8–1.7, *p* = 0.38), suggesting a better fit for the recessive model. Taken together, these data show that the AA genotype of *OAS1* at SNP 10774671 is significantly elevated in individuals seropositive for WNV.

**Table 2:** *OAS1* rs10774671 is associated with increased risk of WNV infection.

	Number	Additive Model			Recessive Model			Dominant Model		
		OR	95% CI	*P*	OR	95% CI	*P*	OR	95% CI	*P*
**All WNV+**	**501**	**1.4**	**1.2–1.6**	**0.0003**	**1.6**	**1.2–2.0**	**0.0002**	**1.5**	**1.0–2.1**	**0.04**
Arizona	135	1.5	1.2–1.9	0.007	1.7	1.2–2.5	0.006	1.6	0.88–2.9	0.12
Colorado	72	1.5	1.1–2.1	0.03	1.5	0.94–2.5	0.08	2.5	0.97–6.3	0.05
California	87	1.2	0.9–1.6	0.36	1.5	0.92–2.3	0.11	0.88	0.49–1.6	0.69
Illinois	37	2	1.2–3.1	0.02	1.7	0.90–3.4	0.11	**	**	0.003*
ARC	170	1.3	1.0–1.7	0.03	1.6	1.1–2.2	0.01	1.2	0.75–2.1	0.4

OR, odds ratio; CI, confidence interval; ARC, American Red Cross. Values were calculated in comparison to the genotype frequency in 552 healthy North American Caucasian WNV-seronegative control samples. Genetic models assume the ancestral G allele as the reference allele. Recessive, GG and GA genotypes combined; Dominant, GA and AA genotypes combined; Additive, GG versus AG and AG versus AA. ORs calculated using JMP software.

*: p value calculated using Fisher's exact test.

**: OR is infinite.

### *OAS1* rs10774671 AA genotype is associated with infection and not severity of clinical outcome

**Figure 3:**
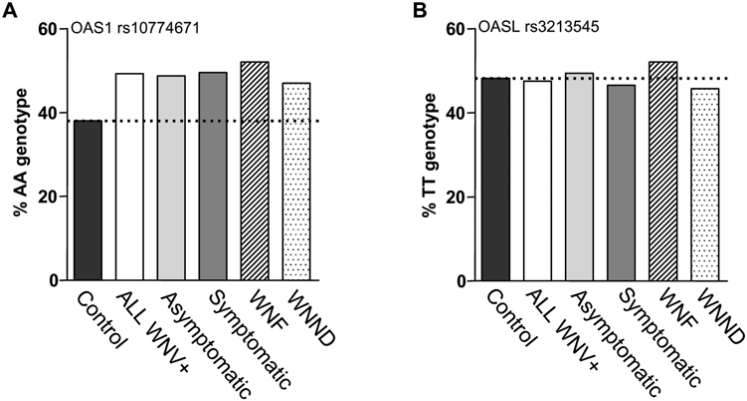
*OAS1* rs10774671 AA genotype frequency is elevated in both symptomatic and asymptomatic WNV-seropositive subjects. (A) A plot of the *OAS1* rs10774671 AA genotypic frequency in all WNV-positive samples (n = 501), WNV-positive samples from individuals who remained symptom-free (asymptomatic; n = 170), and WNV-positive samples from individuals who experienced symptomatic disease (n = 331). Symptomatic WNV-seropositive cases were further examined based on the CDC-defined clinical definition of WNF (n = 119) or WNND (n = 164). Black bar and the dotted line indicate the genotypic frequency of the control population (n = 552). (B) A similar analysis was performed for the *OASL* rs3213545 TT genotype.

We next asked whether the elevated frequency of the A allele in WNV-seropositive subjects varied according to clinical outcome. The AA genotypic frequency for the four groups of symptomatic WNV+ subjects ranged between 47.1–51.1% (average 49.5%, n = 331). The AA genotypic frequency for asymptomatic WNV+ subjects from the ARC fell within this range at 48.8% (n = 170). In contrast, the AA frequency in the control samples was 38.0% (n = 552) (Figure 3A). Analysis of symptomatic patient samples for whom clinical outcome data were available (n = 294) showed a similar distribution of *OAS1* genotypes in both the WNF and WNND clinical subgroups (Table 3 and Figure 3A). With regard to death as an outcome, the sample size is underpowered for analysis: only 13 of all 294 cases for which clinical outcome data were available experienced fatal WNV infection. These data are most consistent with the hypothesis that *OAS1* at SNP 10774671 is a risk factor for seroconversion after exposure to WNV, but not for specific clinical manifestations.

**Table 3:** Distribution of *OAS1* (rs10774671) and *OAS-Like* (rs3213545) genotypes as a function of clinical outcome in symptomatic WNV-seropositive patients.

	*OAS1* AA	*OAS1* AG	*OAS1* GG	*OAS-Like* TT	*OAS-Like* CT	*OAS-Like* CC
WNF^a^	62 (52.1)	40 (33.6)	17 (14.3)	6 (5.0)	51 (42.9)	62 (52.1)
WNND^a^	77 (47.0)	71 (43.3)	16 (9.7)	18 (11.0)	71 (43.3)	75 (45.7)
Not Specified^a^	6 (54.5)	4 (36.4)	1 (9.1)	2 (18.2)	4 (36.4)	5 (45.4)
Death	4 (30.8)	4 (30.8)	5 (38.4)	1 (7.7)	3 (23.1)	9 (69.2)
Total	145 (49.3)	115 (39.1)	34 (11.6)	26 (8.8)	126 (42.9)	142 (48.3)

a Patients were classified into either West Nile fever (WNF), West Nile neuroinvasive disease (WNND), or unspecified disease categories by physician interview. Data do not include the Illinois cases for which no clinical outcome information was provided. No statistically significant differences were observed comparing the genotype frequencies of WNF versus WNND. With regard to death as an outcome, the sample size is underpowered for analysis.

### *OAS1* SNP rs10774671 is independent of *CCR5Δ32* and age

We previously demonstrated that *CCR5Δ32* homozygosity is a strong risk factor for symptomatic WNV disease [20],[21]. To investigate whether the association of *OAS1* SNP 10774671 with WNV infection is independent of *CCR5Δ32*, we analyzed the distribution of *OAS1* in WNV+ subjects from the four groups of symptomatic US Caucasians who were *CCR5+/+* (n = 261), *CCR5+/Δ32* heterozygotes (n = 53), and *CCR5Δ32/Δ32* homozygotes (n = 17). The expected frequencies were calculated solely on *OAS1* HWE considerations, since the increase in *CCR5Δ32* homozygotes resulted in skewing of HWE for this allele, as previously reported [20],[21]. The distribution of *OAS1* genotypes according to *CCR5* genotype did not differ significantly from expectation as shown in Table S1 (χ^2^ = 5.2, *p* = 0.26) suggesting that these alleles assort independently.

Since age is also an accepted risk factor for symptomatic WNV infection, we determined *OAS1* SNP 10774671 genotypes in all symptomatic WNV+ subjects stratified by age; <45 years old (n = 99), between 45 and 64 years old (n = 131), and >64 years old (n = 101) as shown in Table S2, but found no significant differences.

### Lack of *OASL* SNP rs3213545 association with symptomatic WNV disease

We next attempted to validate the association previously identified by Yakub et al. between *OASL* SNP rs3213545 and WNV infection [22]. Contingency analysis (3×2) did not reveal any significant association (*p* = 0.93) when the WNV-infected seropositive subjects (n = 501) were compared to WNV-seronegative controls (n = 552). Analysis of the additive model (OR = 1.0 [95%CI 0.8–1.3], p = 0.94), the recessive model (OR = 1.0, [95%CI 0.69–1.6], p = 0.83), and the dominant model (OR = 1.0, [95%CI 0.8–1.3], p = 0.82) revealed no association with WNV infection (Table S3). When samples collected from each of the 5 centers were analyzed separately, no significant association was found (Table S3). The frequency of TT homozygosity was not elevated above controls (Figure 3B) and did not vary between asymptomatic donors and symptomatic patients. Since the previous study by Yakub et al. showed an association between this SNP and severe WNV disease, we also looked for an association according to clinical outcome. As shown in Table 3 and Figure 3B, no association was found with either WNF or WNND, the more severe CDC clinical definition.

### Development of an ex vivo model for WNV replication in human lymphoid tissue

**Figure 4:**
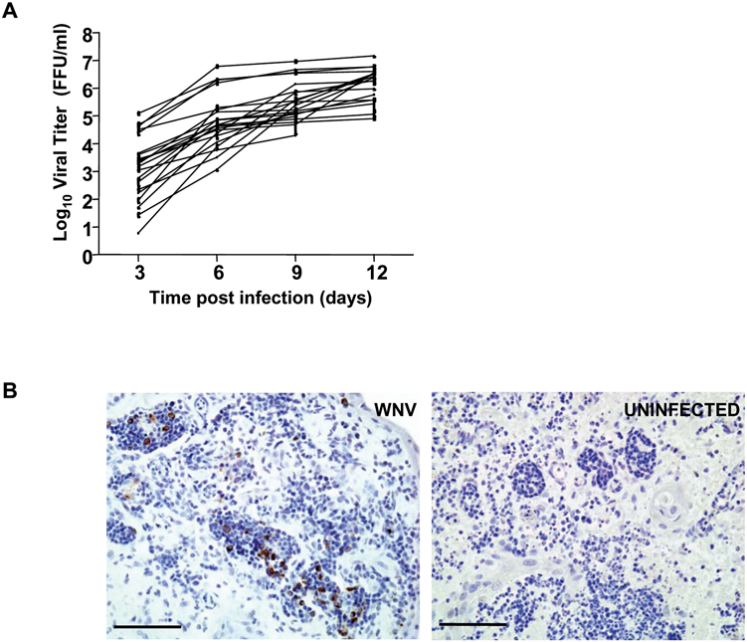
*Ex vivo* model of WNV replication in cultured human lymphoid tissue. (A) Blocks of human tonsil tissue were inoculated *ex vivo* with WNV. Culture supernatant from WNV-infected and -uninfected tonsil tissue was sampled every third day for 12 days and viral titers assessed by FFU assay. 27 tissue blocks were used per experimental condition for each donor. The calculated maximal viral titer based on inoculum alone on day 0 is 1500 FFU/ml. Data represent the mean value of duplicate measurements with standard deviation. Note that data were not assessed for three patients on day 12. (B) Representative WNV immunostaining of WNV-infected (left panel) or donor-matched uninfected lymphoid tissue (right panel) cultured *ex vivo* for nine days. Scale bar, 100 µm.

To directly assess the role of OAS1 during active WNV replication in human cells, we developed an experimental *ex vivo* model of WNV infection using explants of human tonsil tissue. This model may mimic the early events after infection that may occur *in vivo* in the draining lymph node, shown in mice to be an early site of viral replication that precedes CNS spread [30]. Since infection in primary human cells *in vitro* typically requires exogenous stimulation or activation [31],[32], this system is more natural in that the architecture and composition of the primary lymphoid tissue are preserved and no exogenous stimuli are added. Explanted tissue from patients undergoing tonsillectomies was sectioned and inoculated with WNV. For each experimental condition, 27 blocks of tissue were sectioned per donor to control for variation in the number of susceptible cells that may occur between individual slices of tissue from the same donor. Supernatant from the cultured tissue was tested every third day for virus using a focus forming unit (FFU) assay. As shown in Figure 4A, virus was detected in the culture supernatant at the earliest time point (day 3) and increased throughout the 12 day culture for each of the 21 donors tested. WNV-infected cells were visible by direct immunostaining of the tissues (Figure 4B). Tonsils from all donors supported WNV replication to high levels that varied widely among donors, particularly at early time points post-infection (e.g. greater than 4 logs variability at day 3). This model may be useful not only for investigating WNV pathogenesis but also for testing the efficacy of potential WNV therapeutics.

### Early WNV replication may be regulated by *OAS1* genotype

**Figure 5:**
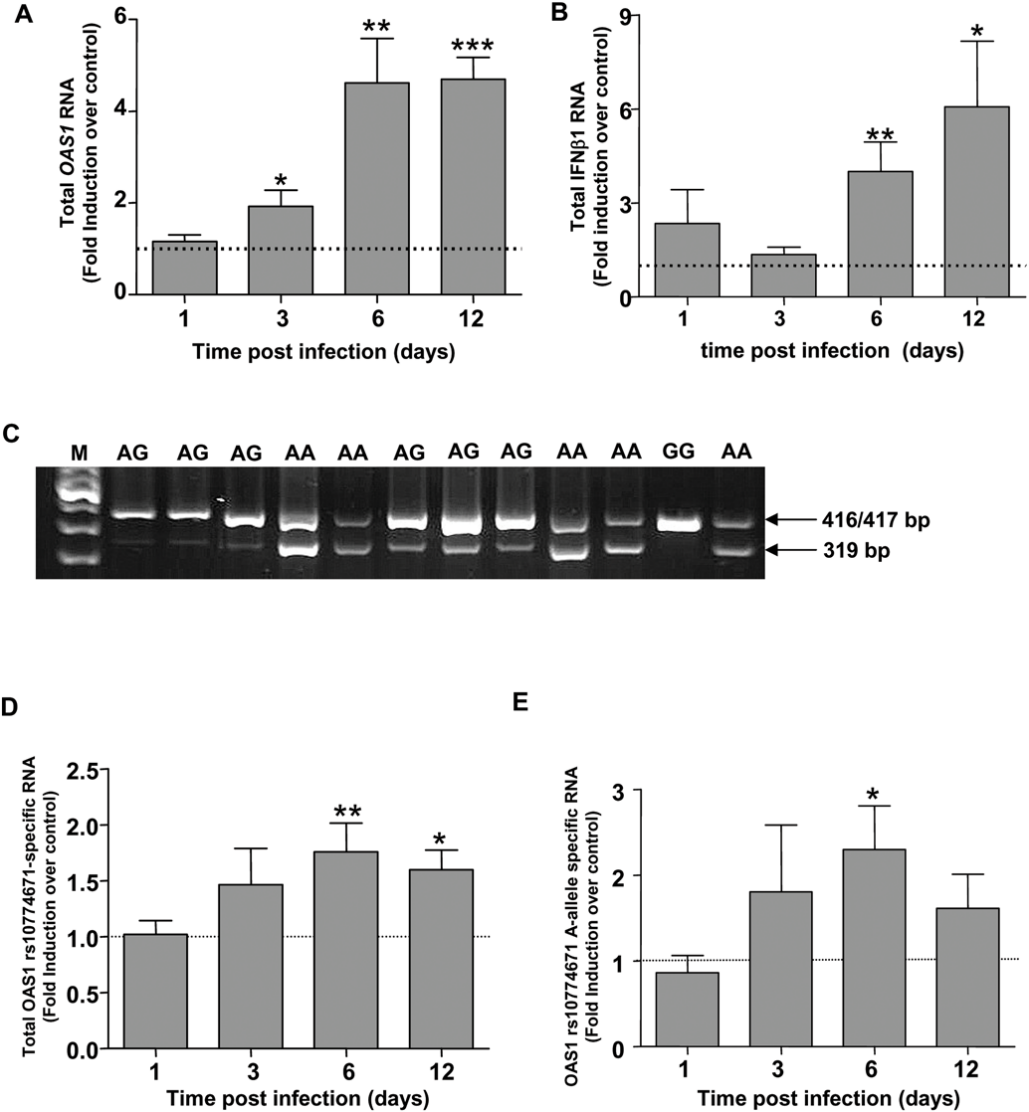
Induction of *OAS1* RNA in human lymphoid tissue infected with WNV. Quantitative RT-PCR analysis of *OAS1* (A) and *IFN-β1* (B) induction in WNV-infected tissue versus matched uninfected tissue taken at various times post-infection from 12 patients and presented as means±SEM. Primers used for *OAS1* analysis are located in exon 2 and 3 and thus amplify all *OAS1* transcripts. (C) *OAS1* genotypes were determined for SNP rs10774671 using ABI SNP genotyping methods. cDNA from uninfected blocks of human tonsil tissue were tested for the presence of *OAS1* SNP-dependent splice variants using primers specifically flanking the SNP as previously described [23]. PCR products were resolved on 2% agarose gel. The presence of the G allele produced an amplicon of 416 bp, while the presence of the A allele gave rise to a 319 bp and 417 bp fragment. Patient genotypes are denoted above the lane; M, molecular weight marker. (D) RNA was extracted at the indicated times after WNV infection of tonsil tissues from 12 donors; uninfected tissue RNA from the same donor served as a control at each time point. cDNA generated was used for PCR using the same primers as described above. Amplicons for the total *OAS1* rs10774671-specific transcripts were quantified using densitometric analysis normalized to *β*-actin. The mean+/−SEM are shown. (E) The induction of *OAS1* rs10774671 A-allele specific RNA from AA homozygous donors was analyzed separately (n = 5). Fold induction is shown as mean+/−SEM. ***p<0.0001; **p<0.005; *p<0.05.

The variation in virus production we observed among donors provided an ideal opportunity to test the dependence of viral replication on *OAS1* genotype in intact human tissue, assuming that OAS1 mRNA was expressed in the tissue. Twelve of the 21 tissues tested for WNV replication were available for RNA extraction to investigate this. First we examined the effect of WNV infection on expression of all OAS1 transcripts (Figure 5A), using primers located between exons 2 and 3. OAS1 mRNA was detected in uninfected tissue from all 12 donors (data not shown), and expression of total OAS1 mRNA was induced ∼2 fold by WNV compared with donor-matched uninfected control tissue at day 3 post-infection (*p* = 0.01; Figure 5A). This increased to ∼4.6 fold induction for both day 6 (*p* = 0.002) and day 12 (*p*<0.0001) after infection. Since OAS1 is an IFN-stimulated gene, we also measured *IFNβ1* in these samples and observed a significant increase above control at day 6 (*p* = 0.002) and day 12 (*p* = 0.007) post infection (Figure 5B). These 12 samples were also tested by RT-PCR for OAS1 rs10774671 A and G allele-specific transcripts using a previously established method [23]. Both A and G allele-specific transcripts (319 and 417 bp amplicons for A allele; 416 bp amplicon for G allele) were detected in appropriate uninfected tissue from all 12 donors (Figure 5C). As shown in Figure 5D, total *OAS1* SNP rs10774671-specific mRNA modestly increased in a time-dependent manner after infection. This was due at least in part to induction of *OAS1* SNP rs10774671 A allele-specific RNA, as shown by analysis of tissue from AA homozygotes (n = 5; Figure 5E). Since RNA generated from the A and G alleles differ by only 1 nucleotide, we were unable to quantitate the relative induction of A and G allele-specific RNA in WNV-infected tissue from AG heterozygotes. We observed ∼1.5-fold induction of G allele-specific mRNA in infected tissue from the one GG homozygote that was available. Thus both *OAS1* SNP rs10774671 variants of interest are present at the RNA level throughout the course of WNV infection of human tonsil tissue, allowing us to ask whether virus replication in this system is *OAS1* genotype-dependent.

**Figure 6:**
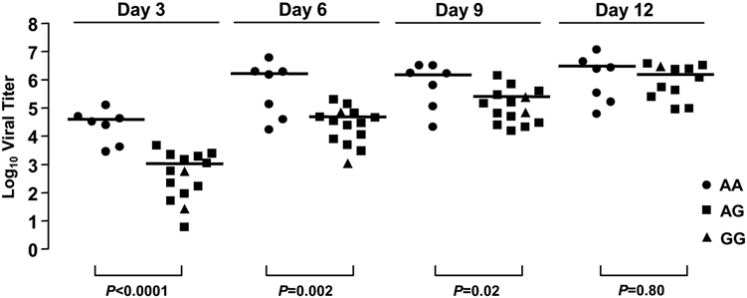
WNV replication of cultured human lymphoid tissue *ex vivo* is *OAS1* genotype-dependent. Viral titers measured at each three-day interval were plotted according to *OAS1* splice acceptor site genotype. Data points represent the amount of virus measured at each three-day interval, and lines indicate the median value. Individuals homozygous for the A allele were compared to AG heterozygotes plus GG homozygotes. Note that viral titers for three patients were not assessed at day 12.

To test whether *OAS1* variation affects WNV infection in human lymphoid tissue *ex vivo*, we genotyped patient samples for the *OAS1* rs10774671 SNP. Of the 21 donors tested, 7 were homozygous for the A allele (33.3%), 2 were homozygous for the G allele (9.5%), and the remaining 12 were AG heterozygotes (57.2%). This distribution is similar to the genotypic distribution observed in controls. When viral production was analyzed as a function of *OAS1* genotype, we found that tissues from individuals homozygous for the A allele supported higher levels than tissues from patients with AG or GG genotypes (Figure 6). The most significant difference was observed 3 days post infection (*p*<0.0001) with a mean viral titer of 3.9×10^4^ ffu in tissues from donors with the AA genotype versus those from individuals who were either AG or GG (1.1×10^3^ ffu). At day 6, the mean viral titer was still greater in AA than in non-AA tissues (1.7×10^6^ versus 5.0×10^4^ ffu, respectively; *p* = 0.002). At day 9, the differences in viral titer had decreased (1.3×10^6^ ffu for AA versus 3.8×10^5^ ffu for non-AA; *p* = 0.02). By day 12 post-infection, no significant difference was observed (*p* = 0.80). Thus the allele associated with higher OAS enzymatic activity (G) is associated with lower viral production for at least 75% of the period of observation after infection in lymphoid tissue.

## Discussion

The present study provides the first evidence in support of the hypothesis that *OAS1* influences risk of infection in response to WNV exposure in humans. This is based on association of the *OAS1* ‘A’ allele at SNP rs10774671, which has previously been associated with low OAS enzymatic activity, with seropositivity for WNV using recessive and additive genetic models on patient samples collected from five US centers. We found that the AA genotype was elevated in WNV-positive individuals regardless of whether these patients remained asymptomatic or developed symptomatic disease, including WNF and WNND. We also observed that homozygosity for the A allele correlated with increased levels of WNV replication in primary lymphoid tissue from human donors. Moreover, the results are consistent with the previously reported identification of the flavivirus resistance locus *flv* in mice as a truncation mutation in *oas1b* [12],[13], the ortholog of human *OAS1* [14]. Our data do not confirm the previously published association of *OASL* and WNV infection in man. Together, these data suggest that OAS1 activity modulates early viral replication, and that decreased OAS1 activity is associated with increased risk of infection and seroconversion. These data suggest that carriers of the G allele, which is associated with high OAS1 activity, may effectively control WNV replication early after infection in humans through innate mechanisms. Furthermore, since *oas1b* in the mouse has been implicated in resistance not only to WNV, but to several other flaviviruses, this association may also be relevant for other medically important neurotropic flaviviruses, such as Japanese encephalitis virus, Yellow Fever virus, and Tick-borne encephalitis virus.

There are several limitations to our study. First, like most gene association studies, the analysis is retrospective. Second, due to a limitation of sample availability the association of WNV infection with *OAS1* SNP 10774671 was determined only in North American Caucasians. Third, the epidemiologic data, while consistent with a role for *OAS1* in controlling WNV infection, do not establish causality versus linkage disequilibrium with an as yet unidentified causal variant. Importantly though, the variant tested has been previously associated with OAS function, and is therefore biologically plausible [23].

Additional work will be needed to delineate the precise mechanisms by which *OAS1* SNP rs10774671 variation may affect WNV infection. We were able to detect the *OAS1* A and G allele transcripts in uninfected human tonsil tissue as predicted by donor genotype, and we found that WNV infection was able to induce total *OAS1* RNA in the tissue as well as RNA specific for the *OAS1* A and G alleles. However, additional work will be needed to determine *OAS1* allele-specific activity in tonsil tissue as a function of WNV infection, and the relative contribution of each of the *OAS1* isoforms present in the tissue. While OAS activity in human PBMCs has been reported to vary in an *OAS1* SNP rs10774671-dependent manner, with the rank order GG>AG>AA, OAS enzymatic activity of purified protein encoded by *OAS1* A and G allele variants has not yet been determined [23]. Furthermore, the mechanism of action of *oas1b* in mice is not yet clear, nor is there evidence that oas1b is enzymatically active [16] or that it functions through activation of RNaseL [15].

Population stratification as a cause of skewed genotypic frequency is a concern in all gene association studies. Given the limited availability of genomic DNA from the small samples of serum available to us from WNV+ subjects, we were unable to address this issue directly. However, we believe this is unlikely to confound our results since the five WNV+ groups were from geographically distinct US populations yet had similar AA genotypic frequencies ranging from 47.1% to 51.4% with an average of 49.3%, whereas the two US WNV-negative control populations that we genotyped, which also were from distinct geographic regions, had much lower AA genotypic frequencies (36.7 and 40.6%) that both fell within a narrow range reported in previously published studies, (36.8–41.1%) [27],[28],[29]. In particular, Smyth et al reported an *OAS1* SNP rs10774671 AA genotypic frequency of 41.1% for 4,735 subjects from the 1958 British Birth Cohort (www.cls.ioe.ac.uk/studies.asp?section=000100020003)[29]; Fedetz et al reported a frequency of 36.8% for 424 Caucasians from Spain [27]; and the International HapMap Project reported a frequency of 39% in US Caucasians (n = 59; www.hapmap.org). It is important to note that a fourth published study, by Field et al., reported an AA genotypic frequency of 49.9% [28]. This anomaly is most likely due to the use of a non-random population, namely the healthy siblings of diabetics. In particular, the A allele frequency in non-transmitted alleles of the parents, which would be expected to have nothing to do with disease, was 0.61 in this study, which would give an expected frequency of 0.61×0.61 = 37% for the AA genotype (L. Field, personal communication), compatible with the frequency found in all five other control populations. Of note, no difference in frequency was observed between cases and controls in a genetically unlinked but related variant in *OASL* (www.hapmap.org), where the odds ratio for all genetic models tested was 1.0, *p*≥0.82.

Animal studies have shown conclusively that the type I IFN system exerts potent antiviral effects critical for survival from viral infections, including WNV infection [9],[30],[33],[34]. However, there is limited evidence in humans supporting a role for endogenous type I IFN signaling at the level of viral pathogenesis. Our study suggests a potentially important role of this type I IFN-inducible pathway at the level of initial infection and containment of virus during the early phases of natural human infection. Our data show that WNV infection is less likely to result in seroconversion in individuals with the GG genotype (higher OAS enzymatic activity), suggesting that individuals with the AA genotype (lower enzymatic activity) are less likely to control initial WNV infection with innate immunity and initiate an adaptive immune response, signaled by seroconversion.

Genetic variation in *OAS1* has been previously associated with other diseases, including Type I Diabetes, SARS, and Hepatitis C. The associations in Type I Diabetes and SARS have been inconsistent in different studies [28],[29],[35],[36]. For Hepatitis C, a relative of WNV also in the flavivirus family, a polymorphism in the 3′-untranslated region of *OAS1* has been associated with persistent infection [37]. This is particularly relevant since this polymorphism is in strong linkage disequilibrium with polymorphism rs10774671 which we have associated with WNV seroconversion [23].

A clinical implication of our results is that pharmacologic induction of *OAS1*, which has been shown to occur in response to IFNα treatment [38],[39] could be efficacious in the context of preventing WNV infection in man. While treatment with IFNα has been reported to be effective in WNV-infected patients, the world experience is limited to a small number of scattered case reports, and treatment failure has also been documented [40],[41],[42]. Our data support continued clinical research of IFNα as a therapeutic option in WNV disease. Finally, our data indicate that the great majority of risk of symptomatic WNV disease remains unexplained at the genetic level, and support continued research into variation in the type I IFN system as a factor contributing to the heterogeneity of outcome in this disease.

## Materials and Methods

### Study populations

The study was approved by the Office of Human Subjects Research of the NIH. Samples of symptomatic WNV-seropositive patient cases from four US states (AZ, CO, CA, and IL) were collected as previously described [20],[21]. Since the allele frequency of this SNP varies by race (www.hapmap.org), and since information about race was not available for all subjects in the WNV-seropositive patient cases, we analyzed only self-reporting Caucasians (total n = 331 in the 4 US states combined). Symptomatic WNV-seropositive patients are defined as individuals who came to clinical attention with symptoms consistent with WNV disease (primarily WNF, WNND), where WNV was confirmed using serological tests. Of the WNV-seropositive samples received, sufficient DNA from self-reported Caucasian patients was available for analysis of 345 samples. Genotypes for both *OAS1* and *OASL* were obtained for 331 symptomatic WNV-seropositive subjects with an overall genotyping success rate of 95.9%. Serum samples were collected from the following states and years: 1) Arizona from the 2004 epidemic (n = 135); 2) Colorado from the 2003 epidemic (n = 72); 3) California from the 2005 epidemic (n = 87); 4) Illinois from the 2005/2006 epidemic (n = 37). Plasma samples from asymptomatic WNV-seropositive blood donors from the American Red Cross (ARC WNV+) were collected between 2003–2008. Asymptomatic WNV-seropositive blood donors (n = 170) are defined as Caucasian random blood donors who tested reactive for WNV nucleic acid twice and were WNV IgM seropositive who remained asymptomatic for at least 2 weeks post donation as assessed by follow-up questionnaire as described previously [25].

Two healthy US Caucasian control groups were established: 1) healthy unrelated US Caucasian random blood donors (RBD) from the NIH Department of Transfusion Medicine (n = 360) collected under an IRB-approved protocol [20]; and 2) healthy Caucasian blood donors from the American Red Cross (n = 192) who tested WNV nucleic acid reactive upon initial screen at the time of blood donation, but were negative for WNV nucleic acid upon retesting and were also WNV-seronegative for IgM (false positives). The following information was provided if available: age, gender, self-reported racial group, date of sample collection, and CDC-defined clinical presentation at the time of sample collection: WNF, WNND, and death. For all study samples, investigators were blinded to unique patient identifiers.

### DNA isolation and genotyping

200 µl of serum collected from Arizona, Colorado, California, and Illinois were thawed for genomic DNA purification using the QiaAmp 96 DNA Blood Kit according to the manufacturer's instructions (Qiagen, Valencia, CA). 250 µl of plasma collected from test subjects by the American Red Cross was purified using NucliSENS EasyMAG automated nucleic acid extraction technology (Biomerieux, Inc). Purified DNA was eluted into 100 µl of the recommended buffer and stored at 4°C until further use. DNA from NIH random blood donors was isolated from peripheral blood leukocytes as previously described [43]. SNPs were genotyped using the ABI 7900HT PCR System with TaqMan primer/probe mix predesigned and validated by ABI (C___2567433_10 and C__11753831_1_). PCR-RFLP was used as a second genotyping method for *OAS1* SNP rs10774671 as previously described [27].

### Statistical analysis

Contingency tables were utilized to calculate genotypic and allelic frequency differences between cases and controls by comparing numbers of expected (Hardy-Weinberg equation) and observed individuals using chi-square tests of significance to obtain a two-sided *p* value using either 1 (2×2 table) or 2 (3×2 table) degrees of freedom. ORs were calculated using a recessive genetic model (i.e. AA verses AG plus GG for *OAS1* and TT versus TC plus CC for *OASL*) or the dominant genetic model (i.e. AA plus AG versus GG for *OAS1* and TT plus TC versus CC for *OASL*) by cross tabulation and 95% CI were estimated using the approximation of Woolf (GraphPad Software version 4.0b, San Diego, CA). The additive model OR values and 95% CI were calculated using JMP software. Chi-square was used unless otherwise indicated. ORs and CIs based on the additive model were estimated using JMP (SAS, version 7). Chi-square goodness-of-fit statistics were used to compare dominant, recessive, and additive models. The two-sided unpaired t-test was used to calculate statistical significance of viral replication after log_10_ transformation of the data for each time point and a two-sided paired t-test was used to calculate significance of gene expression analysis. *P* values were considered significant if <0.05 and the reported values have not been corrected for multiple comparisons. Linkage disequilibrium of *OAS1* SNPs and resultant haplotypes were examined using Haploview 4.0 (available at www.broad.mit.edu/mpg/haploview/index) using previously obtained data from the International HapMap project (www.hapmap.org) on the CEU panel using release 21 data.

### Infection of human lymphoid tissue *ex vivo*

Human tonsils from routine tonsillectomy performed at the Children's Hospital National Medical Center in Washington, DC were collected under an Institutional Review Board-approved protocol from NICHD within a few hours after surgery. The mean age of the children used in this study was 5±3. Tonsils were dissected in blocks of approximately 2 mm. 9 tissue blocks per well in triplicate wells per condition were placed on top of collagen sponge gels floating in six-well plates as previously described [44]. All 27 tissue blocks from each donor were then individually inoculated with 5 µl containing 500 FFU WNV strain NY99, or left uninfected. We assessed productive WNV infection by measuring FFU as described above in culture medium pooled from 27 blocks during the previous 3 days between successive media changes. FFU at each time point represent the cumulative total of infectious virions produced. Coefficient of variation for each donor and each time point was calculated to be 13.3%. All tonsil donors were analyzed for the *CCR5Δ32* mutation as previously described [20] and found to be wild type at this allele.

### Viral Titers

Confluent Vero cells were grown in a 12 well plate in OptiPro SFM (Invitrogen) with 2% FBS (Hyclone) and 50 µg/ml gentamicin (ATCC). Supernatants from tonsil cultures were diluted and incubated on the cells for 1 hour at 37°C prior to overlaying with 2 ml Opti-MEM (Invitrogen) with 8 g/L methylcellulose (Sigma), 2% FBS and 50 µg/ml gentamicin. Plates were incubated for 2 days at 37°C, then washed 3 times with PBS. 500 µl of diluted anti-WNV anti-sera/well (HMAF, ATCC #VR-82) was incubated for 1 hour at 37°C. Plates were then washed 3 times and 500 µl of diluted (1∶10) anti-mouse/anti-rabbit HRP labeled polymer (DAKO Cytomation) was added. Cells were incubated for 1 hour at 37°C, washed 3 times and focus forming units (FFU) of WNV were visualized by addition of 1 ml diaminobenzidine (DAB) mixture (4.5 mg DAB (Sigma) /10 ml PBS+4.5 µl 30% H_2_0_2_ /10 ml). Viral titers are expressed as FFU/ml.

### Real-time PCR

RNA was extracted from lymphoid tissue using the RNeasy Tissue kit according to manufacturer's protocol (Qiagen). Reverse transcription was performed using Superscript III first strand synthesis Supermix (Invitrogen) with random hexamers. *OAS1* (Hs00242943_m1), IFNβ1 (Hs00277188_s1) and *GAPDH* (Hs00266705_g1) primer/probe sets were obtained from ABI and cycled on an ABI 7900HT PCR System. Each sample was normalized to GAPDH and fold change in infected samples were compared to uninfected donor-matched samples.

### Immunohistochemistry

4–5 µm sections of paraffin-embedded slides were stained with anti-WNV hyperimmune mouse ascites fluid (HMAF, ATCC #VR-82) diluted 1∶100 in antibody diluent, background reducing agent (DAKO Cytomation). After incubation with anti-mouse polymer-horse radish peroxidase (DAKO Cytomation), slides were developed with streptavidin and diaminobenzidine liquid (DAKO Cytomation) and counterstained with hematoxylin.

### Detection of *OAS1* splice variants

Tissues from 12 tonsil donor samples were tested for OAS1 SNP-specific mRNA. cDNA from uninfected and infected donor-matched samples for each time point amplified by PCR as previously described [23]. Briefly, primers were designed to flank the splice acceptor site (F-ggcggaccctacaggaaact; R-acaccagctcactgaggagc). The presence of a G allele resulted in a 416 bp amplicon, while the presence of the A allele resulted in 319 bp and 417 bp fragments. PCR products were resolved on a 2% agarose gel, visualized using ethidium bromide, and quantitated by densitometry normalized to β-actin using Image J software version 1.38×. Fold change was calculated by comparing normalized WNV-infected measurements to normalized uninfected measurements for each time point for each donor.

## Supporting Information

Table S1 Distribution of *OAS1* (rs10774671) and *CCR5Δ32* genotypes in symptomatic WNV-seropositive patients
(0.03 MB DOC)

Table S2 Distribution of *OAS1* (rs10774671) genotypes according to age in Caucasian symptomatic WNV-seropositive patient samples
(0.03 MB DOC)

Table S3 *OAS-Like* SNP rs3213545 is associated with increases risk of WNV infection
(0.04 MB DOC)

## References

[1] Smithburn KC, Hughes TP, Burke AW, Paul JH (1940). A neurotropic virus isolated from the blood of a native of Uganda.. Am J Trop Med.

[2] Asnis DS, Conetta R, Teixeira AA, Waldman G, Sampson BA (2000). The West Nile Virus outbreak of 1999 in New York: the Flushing Hospital experience.. Clin Infect Dis.

[3] Hubalek Z, Halouzka J (1999). West Nile fever—a reemerging mosquito-borne viral disease in Europe.. Emerg Infect Dis.

[4] Lanciotti RS, Roehrig JT, Deubel V, Smith J, Parker M (1999). Origin of the West Nile virus responsible for an outbreak of encephalitis in the northeastern United States.. Science.

[5] Mostashari F, Bunning ML, Kitsutani PT, Singer DA, Nash D (2001). Epidemic West Nile encephalitis, New York, 1999: results of a household-based seroepidemiological survey.. Lancet.

[6] Hayes EB, Gubler DJ (2006). West Nile virus: epidemiology and clinical features of an emerging epidemic in the United States.. Annu Rev Med.

[7] Samuel MA, Whitby K, Keller BC, Marri A, Barchet W (2006). PKR and RNase L contribute to protection against lethal West Nile Virus infection by controlling early viral spread in the periphery and replication in neurons.. J Virol.

[8] Hovanessian AG, Justesen J (2007). The human 2′–5′oligoadenylate synthetase family: unique interferon-inducible enzymes catalyzing 2′–5′ instead of 3′–5′ phosphodiester bond formation.. Biochimie.

[9] Samuel MA, Diamond MS (2005). Alpha/beta interferon protects against lethal West Nile virus infection by restricting cellular tropism and enhancing neuronal survival.. J Virol.

[10] Sangster MY, Heliams DB, MacKenzie JS, Shellam GR (1993). Genetic studies of flavivirus resistance in inbred strains derived from wild mice: evidence for a new resistance allele at the flavivirus resistance locus (Flv).. J Virol.

[11] Sangster MY, Urosevic N, Mansfield JP, Mackenzie JS, Shellam GR (1994). Mapping the Flv locus controlling resistance to flaviviruses on mouse chromosome 5.. J Virol.

[12] Mashimo T, Lucas M, Simon-Chazottes D, Frenkiel MP, Montagutelli X (2002). A nonsense mutation in the gene encoding 2′–5′-oligoadenylate synthetase/L1 isoform is associated with West Nile virus susceptibility in laboratory mice.. Proc Natl Acad Sci U S A.

[13] Perelygin AA, Scherbik SV, Zhulin IB, Stockman BM, Li Y (2002). Positional cloning of the murine flavivirus resistance gene.. Proc Natl Acad Sci U S A.

[14] Mashimo T, Glaser P, Lucas M, Simon-Chazottes D, Ceccaldi PE (2003). Structural and functional genomics and evolutionary relationships in the cluster of genes encoding murine 2′,5′-oligoadenylate synthetases.. Genomics.

[15] Scherbik SV, Paranjape JM, Stockman BM, Silverman RH, Brinton MA (2006). RNase L plays a role in the antiviral response to West Nile virus.. J Virol.

[16] Rogozin IB, Aravind L, Koonin EV (2003). Differential action of natural selection on the N and C-terminal domains of 2′–5′ oligoadenylate synthetases and the potential nuclease function of the C-terminal domain.. J Mol Biol.

[17] Kajaste-Rudnitski A, Mashimo T, Frenkiel MP, Guenet JL, Lucas M (2006). The 2′,5′-oligoadenylate synthetase 1b is a potent inhibitor of West Nile virus replication inside infected cells.. J Biol Chem.

[18] Lucas M, Mashimo T, Frenkiel MP, Simon-Chazottes D, Montagutelli X (2003). Infection of mouse neurones by West Nile virus is modulated by the interferon-inducible 2′–5′ oligoadenylate synthetase 1b protein.. Immunol Cell Biol.

[19] Scherbik SV, Kluetzman K, Perelygin AA, Brinton MA (2007). Knock-in of the Oas1b(r) allele into a flavivirus-induced disease susceptible mouse generates the resistant phenotype.. Virology.

[20] Glass WG, McDermott DH, Lim JK, Lekhong S, Yu SF (2006). CCR5 deficiency increases risk of symptomatic West Nile virus infection.. J Exp Med.

[21] Lim JK, Louie CY, Glaser C, Jean C, Johnson B (2007). Genetic deficiency of chemokine receptor CCR5 is a strong risk factor for symptomatic West Nile Virus infection: a meta-analysis of 4 cohorts in the US epidemic.. J Infect Dis.

[22] Yakub I, Lillibridge KM, Moran A, Gonzalez OY, Belmont J (2005). Single nucleotide polymorphisms in genes for 2′–5′-oligoadenylate synthetase and RNase L inpatients hospitalized with West Nile virus infection.. J Infect Dis.

[23] Bonnevie-Nielsen V, Field LL, Lu S, Zheng DJ, Li M (2005). Variation in antiviral 2′,5′-oligoadenylate synthetase (2′5′AS) enzyme activity is controlled by a single-nucleotide polymorphism at a splice-acceptor site in the OAS1 gene.. Am J Hum Genet.

[24] Justesen J, Hartmann R, Kjeldgaard NO (2000). Gene structure and function of the 2′–5′-oligoadenylate synthetase family.. Cell Mol Life Sci.

[25] Orton SL, Stramer SL, Dodd RY (2006). Self-reported symptoms associated with West Nile virus infection in RNA-positive blood donors.. Transfusion.

[26] Stramer SL, Fang CT, Foster GA, Wagner AG, Brodsky JP (2005). West Nile virus among blood donors in the United States, 2003 and 2004.. N Engl J Med.

[27] Fedetz M, Matesanz F, Caro-Maldonado A, Fernandez O, Tamayo JA (2006). OAS1 gene haplotype confers susceptibility to multiple sclerosis.. Tissue Antigens.

[28] Field LL, Bonnevie-Nielsen V, Pociot F, Lu S, Nielsen TB (2005). OAS1 splice site polymorphism controlling antiviral enzyme activity influences susceptibility to type 1 diabetes.. Diabetes.

[29] Smyth DJ, Cooper JD, Lowe CE, Nutland S, Walker NM (2006). No evidence for association of OAS1 with type 1 diabetes in unaffected siblings or type 1 diabetic cases.. Diabetes.

[30] Bourne N, Scholle F, Silva MC, Rossi SL, Dewsbury N (2007). Early production of type i interferon during West Nile virus infection: role for lymphoid tissues in IRF3-independent interferon production.. J Virol.

[31] Pierson TC, Diamond MS, Ahmed AA, Valentine LE, Davis CW (2005). An infectious West Nile virus that expresses a GFP reporter gene.. Virology.

[32] Rios M, Zhang MJ, Grinev A, Srinivasan K, Daniel S (2006). Monocytes-macrophages are a potential target in human infection with West Nile virus through blood transfusion.. Transfusion.

[33] Levy DE, Garcia-Sastre A (2001). The virus battles: IFN induction of the antiviral state and mechanisms of viral evasion.. Cytokine Growth Factor Rev.

[34] Munoz-Jordan JL, Laurent-Rolle M, Ashour J, Martinez-Sobrido L, Ashok M (2005). Inhibition of alpha/beta interferon signaling by the NS4B protein of flaviviruses.. J Virol.

[35] Hamano E, Hijikata M, Itoyama S, Quy T, Phi NC (2005). Polymorphisms of interferon-inducible genes OAS-1 and MxA associated with SARS in the Vietnamese population.. Biochem Biophys Res Commun.

[36] He J, Feng D, de Vlas SJ, Wang H, Fontanet A (2006). Association of SARS susceptibility with single nucleic acid polymorphisms of OAS1 and MxA genes: a case-control study.. BMC Infect Dis.

[37] Knapp S, Yee LJ, Frodsham AJ, Hennig BJ, Hellier S (2003). Polymorphisms in interferon-induced genes and the outcome of hepatitis C virus infection: roles of MxA, OAS-1 and PKR.. Genes Immun.

[38] Kim KI, Kim SR, Sasase N, Taniguchi M, Harada S (2006). 2′-,5′-Oligoadenylate synthetase response ratio predicting virological response to PEG-interferon-alpha2b plus ribavirin therapy in patients with chronic hepatitis C.. J Clin Pharm Ther.

[39] Murashima S, Kumashiro R, Ide T, Miyajima I, Hino T (2000). Effect of interferon treatment on serum 2′,5′-oligoadenylate synthetase levels in hepatitis C-infected patients.. J Med Virol.

[40] Kalil AC, Devetten MP, Singh S, Lesiak B, Poage DP (2005). Use of interferon-alpha in patients with West Nile encephalitis: report of 2 cases.. Clin Infect Dis.

[41] Lewis M, Amsden JR (2007). Successful treatment of West Nile virus infection after approximately 3 weeks into the disease course.. Pharmacotherapy.

[42] Chan-Tack KM, Forrest G (2005). Failure of interferon alpha-2b in a patient with West Nile virus meningoencephalitis and acute flaccid paralysis.. Scand J Infect Dis.

[43] Zimmerman PA, Buckler-White A, Alkhatib G, Spalding T, Kubofcik J (1997). Inherited resistance to HIV-1 conferred by an inactivating mutation in CC chemokine receptor 5: studies in populations with contrasting clinical phenotypes, defined racial background, and quantified risk.. Mol Med.

[44] Glushakova S, Baibakov B, Margolis LB, Zimmerberg J (1995). Infection of human tonsil histocultures: a model for HIV pathogenesis.. Nat Med.

