# Supplementary material for: Genetic Variation in OAS1 Is a Risk Factor for Initial Infection with West Nile Virus in Man
Source: PLoS Pathog. 2009 Feb 27;5(2):e1000321. doi: 10.1371/journal.ppat.1000321 (PMC2642680; doi:10.1371/journal.ppat.1000321)
Supplement: Table S1 — Distribution of OAS1 (rs10774671) and CCR5Δ32 genotypes in symptomatic WNV-seropositive patients (0.03 MB DOC) [file ppat.1000321.s001.doc]

Table S1. Distribution of *OAS1* (rs10774671) and *CCR5Δ32* genotypes in symptomatic WNV seropositive patients.

|  | | ***CCR5+/CCR5+*** | | ***CCR5+/Δ32*** | | ***Δ32/Δ32*** | |
| --- | --- | --- | --- | --- | --- | --- | --- |
|  | | **observed** | **expected** | **observed** | **expected** | **observed** | **expected** |
| *OAS1* AA | | 127 | 127 | 25 | 26 | 12 | 8 |
| *OAS1* AG | | 109 | 110 | 21 | 22 | 3 | 7 |
| *OAS1* GG | | 25 | 24 | 7 | 5 | 2 | 2 |
|  | CCR5+=wild type allele; *Δ32*=CCR5 delta 32 mutant allele; Expected values were determined based solely on *OAS1* HWE calculations. χ^2^=5.2, *P*=0.26. | | | | | | |
