# Supplementary material for: Genetic Variation in OAS1 Is a Risk Factor for Initial Infection with West Nile Virus in Man
Source: PLoS Pathog. 2009 Feb 27;5(2):e1000321. doi: 10.1371/journal.ppat.1000321 (PMC2642680; doi:10.1371/journal.ppat.1000321)
Supplement: Table S2 — Distribution of OAS1 (rs10774671) genotypes according to age in Caucasian symptomatic WNV-seropositive patient samples (0.03 MB DOC) [file ppat.1000321.s002.doc]

Table S2. Distribution of *OAS1* (rs10774671) genotypes according to age in Caucasian symptomatic WNV seropositive patient samples.

| Genotype | <45 years old | 45-64 years old | >64 years old |
| --- | --- | --- | --- |
| *OAS1* AA (%) | 49 (49.5%) | 67 (51.1%) | 48 (47.5%) |
| *OAS1* AG (%) | 40 (40.4%) | 49 (37.4%) | 44 (43.6%) |
| *OAS1* GG (%) | 10 (10.1%) | 15 (11.5%) | 9 (8.9%) |
| Total # patients | 141 | 223 | 137 |
