# Supplementary material for: Genetic Variation in OAS1 Is a Risk Factor for Initial Infection with West Nile Virus in Man
Source: PLoS Pathog. 2009 Feb 27;5(2):e1000321. doi: 10.1371/journal.ppat.1000321 (PMC2642680; doi:10.1371/journal.ppat.1000321)
Supplement: Table S3 — OAS-Like SNP rs3213545 is associated with increases risk of WNV infection (0.04 MB DOC) [file ppat.1000321.s003.doc]

Table S3. *OAS-Like* SNP rs3213545 is associated with increases risk of WNV infection.

|  |  |  | Additive Model | | |  | Recessive Model | | |  | Dominant Model | | |
| --- | --- | --- | --- | --- | --- | --- | --- | --- | --- | --- | --- | --- | --- |
| Cohort |  | n | OR | 95% CI | *P* |  | OR | 95% CI | *P* |  | OR | 95% CI | *P* |
| **All WNV+** |  | **501** | **1.0** | **0.8-1.3** | **0.94** |  | **1.0** | **0.69-1.6** | **0.83** |  | **1.0** | **0.81-1.3** | **0.82** |
| Arizona |  | 135 | 1.1 | 0.8-1.6 | 0.52 |  | 1.5 | 0.75-3.2 | 0.24 |  | 0.97 | 0.67-1.4 | 0.88 |
| Colorado |  | 72 | 0.8 | 0.5-1.2 | 0.11 |  | 0.61 | 0.31-1.2 | 0.17 |  | 1.4 | 0.84-2.3 | 0.21 |
| California |  | 87 | 1.2 | 0.8-2.0 | 0.24 |  | 1.5 | 0.62-3.6 | 0.37 |  | 0.79 | 0.50-1.2 | 0.31 |
| Illinois |  | 37 | 0.7 | 0.4-1.2 | 0.14 |  | 0.91 | 0.31-2.7 | 0.87 |  | 1.9 | 0.95-3.9 | 0.06 |
| ARC |  | 170 | 1.0 | 0.7-1.4 | 0.92 |  | 0.93 | 0.53-1.6 | 0.81 |  | 0.95 | 0.67-1.3 | 0.78 |
| OR =odds ratio, CI =confidence interval, ARC=American Red Cross. Values were calculated in comparison to the genotype frequency in 552 North American Caucasian WNV seronegative control samples. Genetic models assume the major C allele as the reference allele. Recessive, CC and CT genotypes combined; Dominant, CT and TT genotypes combined; Additive, CC versus CT and CT versus TT. ORs calculated using JMP software. | | | | | | | | | | | | | |
